# Study of genetic variation and its association with tensile strength among bamboo species through whole genome resequencing

**DOI:** 10.3389/fpls.2022.935751

**Published:** 2022-07-27

**Authors:** Lorenzo Del Giudice, Christos Bazakos, Michalis F. Vassiliou

**Affiliations:** ^1^Chair of Seismic Design and Analysis, Institute of Structural Engineering, ETH Zurich, Zurich, Switzerland; ^2^Institute of Plant Breeding and Genetic Resources, ELGO-Dimitra, Thessaloniki, Greece; ^3^Joint Laboratory of Horticulture, ELGO-Dimitra, Thessaloniki, Greece; ^4^Department of Comparative Development and Genetics, Max Planck Institute for Plant Breeding Research, Cologne, Germany

**Keywords:** structural engineering, construction materials, bamboo, genetic variation, genome resequencing

## Abstract

Moso bamboo (*Phyllostachys edulis*) is a versatile plant species that is widely used as a construction material by many low-income countries due to the lack of major construction materials such as steel and reinforced concrete. It is also widely used in China. Bamboo is an economically sustainable material that behaves exceptionally in natural disasters such as earthquakes and it can offer viable solutions for contemporary engineering challenges. Despite bamboo’s potential in the engineering sector, biological features such as its long generation time, its large genome size, and its polyploidy are constraining factors for genetic and genomic studies that potentially can assist the breeding efforts. This study re-sequenced 8 *Phyllostachys* species and 18 natural accessions of *Ph. edulis*, generating a large set of functionally annotated molecular markers (Single Nucleotide Polymorphisms (SNPs) and InDels) providing key genomic resource information. Moreover, all this genomic information was used to carry out a preliminary genome-wide association analysis and several candidate genes were identified to be correlated with a mechanical property that is of high interest to structural engineers: its tensile strength normal to its fibers (i.e., splitting).

## Introduction

Modern civil engineering was mainly developed in places where the main construction materials are Steel and Reinforced Concrete (RC). However, it so happens that many underdeveloped countries do not have steel and RC but do have more sustainable materials that are also of lower cost. These materials have been developed less, not because they are inherently insufficient, but because they have not received the attention of contemporary engineering. Bamboo is such a material. It has been used for centuries in Southeast Asia, Africa, and Latin America and has behaved exceptionally in natural disasters such as earthquakes (Vengala et al., 2015). Therefore, improving the mechanical properties of the bamboo species that are used in construction, both in terms of increasing their average values and in terms of reducing their variability, could have a large impact on the construction industry.

Part of the variability of the bamboo’s mechanical properties, both within a species and along different bamboo species can be attributed to its genetic background (Zhao et al., 2021). The Moso Bamboo genome was recently sequenced and assembled at the chromosome level (Zhao et al., 2018). The bamboo genome is diploid and contains 24 chromosomes of a total size of 2.05 Gb, encoding (at least) 31,987 genes (Peng et al., 2013). Unlike in other plants, in which generating a synthetic population by performing crosses of selected strains is a feasible and cost-effective way to perform genotype to phenotype associations, creating a synthetic population of bamboo is a challenge. In particular, most species and strains of bamboo can remain in the vegetative phase for decades before flowering (i.e., reproductive) phase, making the crossing in the laboratory decidedly impractical (Ramakrishnan et al., 2020). Interestingly, some strains flower every year that can potentially assist the breeding efforts.

Despite the high importance of bamboo for its construction properties, features such as large genome size, polyploidy, and long generation time are limiting factors for genetic and genomic research. A recent genome-wide study on the LTR-retrotransposons elements assessed the genetic variability and population structure of Asian bamboo by developing IRAP-based markers (Li et al., 2020). Genome Wide Association Study (GWAS) has been proved to be a powerful tool in detecting phenotype–genotype relationships and dissecting the genetic architecture of complex quantitative traits in model and crop plants (Huang and Han, 2014; Bazakos et al., 2017). GWASs examine the genetics of natural variation by studying natural populations and taking advantage of their long history of recombination events to identify haplotype blocks associated with phenotypes of interest (Bazakos et al., 2017). With the rapid development of sequencing technologies and computational methods, GWAS usage spread and became a common method for detecting natural variation of complex traits in model plants and crops (Huang and Han, 2014). In this study, a panel comprising 8 *Phyllostachys* species and 18 natural accessions of *Phyllostachys edulis* (*Ph. edulis*) were used to carry out a genome-wide association analysis for a mechanical property, which is of high interest to structural engineers: its tensile strength normal to its fibers (i.e., splitting).

## Materials and methods

### Plant material and description of collection site

The bamboo samples used in the present study were collected in Anduze, south France, where there are plantations of different bamboo species. The plantation from which we collected the samples is divided into four sites (S1–S4) where different rhizomes/genotypes of *Phyllostachys* species are grown. The specimens were collected in September 2019. The following species were collected: *Phyllostachys edulis* (*Ph_edulis*), *Phyllostachys nigra* “Boryana” (Ph_nigra_boryana), *Phyllostachys dulcis* (Ph_dulcis), *Phyllostachys bambusoides* (Ph_bambusoides), *Ph. bambusoides* “Holochrysa” (Ph_bambusoides_holochrysa), *Phyllostachys viridiglaucescens* (Ph_Viridiglaucescens), *Phyllostachys nigra* “Henonis” (Ph_Nigra_Henonis), and *Ph. bambusoides* “Castillonis” (Ph_Bambusoides_Castillonis). In total, 25 culms with their leaves were collected. The age of the culms was between 3 and 5 years. It was not possible to determine it with higher accuracy. Table 1 reports the details of the number of samples per species, as well as the site and area from which the culms were harvested. Three specimens were taken from each culm (bottom, middle, and top) in accordance with ISO 22157:2019, 2019, hence the total number of specimens was 75.

**TABLE 1:** Effects of the identified SNPs on genes.

Impact class	Count	Percent (%)
High	63,341	0.036
Moderate	1,027,868	0.577
Low	1,318,370	0.741
Modifier	175,603,997	98.646

### Mechanical tests

**FIGURE 1:**
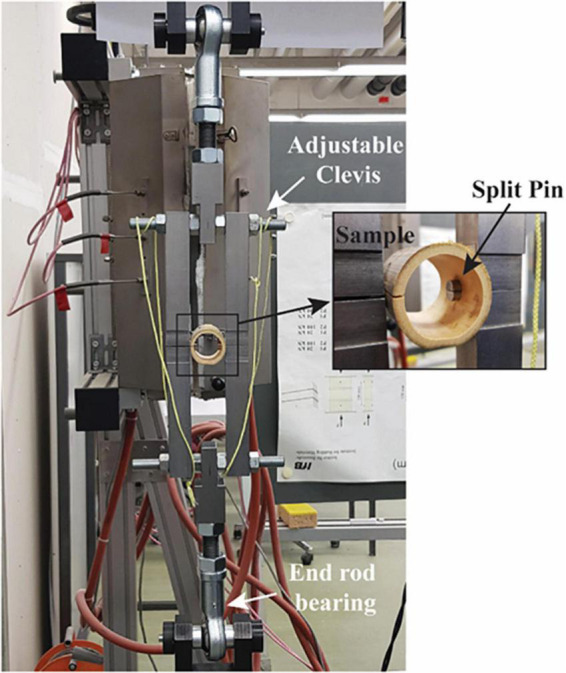
Testing apparatus and details of specimen mounting.

**FIGURE 2:**
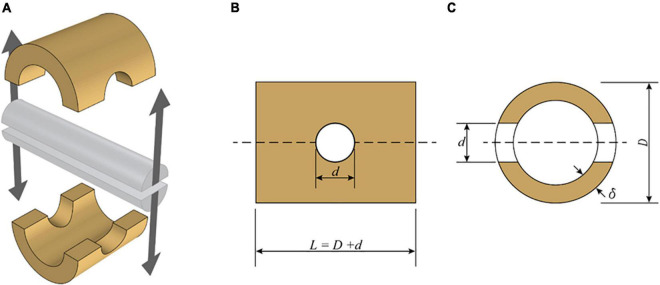
Schematic view of split-pin apparatus modified from ISO 2019. **(A)** Split-pin apparatus. **(B)** Side elevation of a specimen. **(C)** Section through the specimen.

The testing apparatus consists of a split pin assembly (Figure 1) mounted in a universal testing machine (UTM). Each half of the split pin is connected to the UTM through an adjustable clevis with a rod end bearing. The geometric flexibility of the clevis is necessary to accommodate specimens of different geometries. The specimens were mounted to the testing apparatus by inserting the split pin in a hole drilled normal to the culm length. The dimensions of each bamboo specimen (Figure 2), that is, the length (*L*) and the diameter of the drilled hole (*d*), are defined in ISO 221757:2019, and they are a function of the external diameter *D* and wall thickness of each bamboo sample, δ: The diameter of the drilled hole should range between 0.25 and 0.50 *D*, and the length must be equal to *L* = *D* + *d.*

The tests were carried out in displacement control after having applied a preload of 5N which is necessary to ensure that there is no gap between the split-pin and the sample. The test velocity is not explicitly declared in the ISO 221757:2019. Nonetheless, the standard prescribes that speed should be such that fracture would occur after 300 ± 120 s.

The splitting strength (*f*_*t*,90_) is calculated according to ISO 221757:2019, with the following formula


(1) $$f_{t,90}=\frac{F_{u\,l\,t}}{2\,\delta \,\left(L-\delta \right)}.$$

where *F*_*ult*_ is the maximum load reached during the splitting test, δ is the wall thickness of the specimen, *d* is the diameter of the split-pin, and *L* is the length of the specimen (Figure 2).

Due to the strong influence that the moisture content has on the strength of bamboo (Hone et al., 2020), all samples were stored in a climate chamber at 65% relative humidity and 25°C for at least a month. This prevented cracking of the bamboo samples due to variation in the moisture content of the bamboo, and it homogenized the moisture content across different samples.

#### Specimen preparation

The split pin test requires the preparation of each sample according to ISO 221757:2019 as shown in Figure 1. As the culm cross-section is not a perfect circle, *D* was set equal to the average between two measurements taken perpendicularly to each other. Two different diameters were chosen for the drilled hole diameter *d* (12 and 22 mm). These two dimensions were chosen such that *d* would be in the range of 0.25–0.5 *D* for all specimens. The outer diameter *D* of the tested specimens ranged between 20.3 and 102.3 mm, the wall thickness, δ between 1.5 and 12 mm, and the specimen length *L* between 32.3 and 124.3 mm (the dimensions of all specimens are reported in Appendix Table A1).

Principal component analysis (PCA) in a biplot was performed using the package “ggbiplot” in R and hierarchical cluster analysis (HCA), and heatmap representation were performed using the software ClustVis (Metsalu and Vilo, 2015).

### DNA isolation and whole genome re-sequencing

High-quality DNA was extracted from young leaves from bamboo culm of each genotype using NucleoSpin Plant II Mini kit (Macherey–Nagel) according to the manufacturer’s instructions. After dilution to 100 ng/μl, the 25 genomic DNA samples were used to generate 25 Tru-Seq Nano libraries with a mean insertion size of 450 bp and the libraries were sequenced, Paired-End 150 bp, using an Illumina NovaSeq S4 system (Illumina, San Diego, CA, United States).

### Read quality controls, alignment, and post alignment quality controls

Quality control, pre-processing, and alignment of Illumina paired-end (PE) reads were performed using the workflow manager SUSHI (Hatakeyama et al., 2016) developed at the Functional Genomics Center, Zurich (FGCZ). In detail, technical quality was evaluated using FastQC (v 0.11.9) and MultiQC (v 1.9). Possible cross-sample contaminations were screened using FastqScreen (v 0.14.1) against a customized database, which consists of SILVA rRNA sequences,^1^ UniVec,^2^ refseq mRNA sequences and selected genome sequences (human, mouse, arabidopsis, bacteria, virus, phix, lambda, and mycoplasma).^3^ The raw paired-end sequence data reported in FASTQ format was deposited in the National Centre for Biotechnology Information’s (NCBI) Short Read Archive (SRA) database under the accession number PRJNA817562. Illumina PE reads were pre-processed using fastp (v 0.20), where sequencing adapters and low-quality ends (<Q20) were trimmed. Trimmed reads passing the filtering criteria (average quality ≥ Q20, minimum length ≥ 18 bp) were aligned to the bamboo *Ph. edulis* reference genome^4^ (Zhao et al., 2018) using bwa mem (v 0.7.17). PCR duplicates were marked using Picard (v 2.22.8). Read alignments were comprehensively evaluated using the mapping QC app in SUSHI, in terms of different aspects of genomic DNA sequencing experiments, such as mapping quality, sequencing depth, and coverage uniformity.

### Analysis of single nucleotide polymorphisms and small InDels

Multi-sample joint variant calling was performed using samtools/bcftools (v1.11) (Li et al., 2009). Identified small variants were further classified as Single Nucleotide Polymorphisms (SNPs) only or InDels only using vcftools (v0.1.16).^5^ Using the same tools, variants were filtered with variant quality Q20 and above. Variants belonging to individual samples were extracted using bcftools (v1.11). Variants were annotated with reference gene models (see text footnote 4) using SnpEff (v 4.3) (Cingolani et al., 2012).

### Genome wide association analysis using small variants

The reference genome has thousands of scaffolds rather than only a few chromosomes; therefore, a map file that defines every scaffold as one “chromosome” was first generated from the input filtered (Q20 and above) vcf file using bcftools (v1.11) and awk (v4.1.1) (bcftools view -H freebayes.q20.vcf.gz | cut -f 1 | uniq | awk ‘{print $0″\t”$0}’ > chrom-map.txt). In the map file, the scaffold IDs were used also as chromosome IDs for simplicity.

VCFTools (v0.1.16) was then used to convert the vcf file and the map file to PLINK format map and ped files (vcftools –gzvcf freebayes.q20.vcf.gz –plink –chrom-map chrom-map.txt –out freebayes.q20). The PLINK map and ped files were finally converted to PLINK binary files using PLINK (v1.90b6.21, –allow-extra-chr –allow-no-sex –make-bed –noweb) (Chang et al., 2015).

Association analysis was performed with the PLINK binary files (–bfile) and a file of quantitative traits (–pheno) using the default Wald test, reporting multiple-testing corrections and values for Q-Q plot (–adjust qq-plot). Only bi-allelic variants with MAF above 0.05, with variant pruning, were included in the association analysis (–assoc –maf 0.05 –indep-pairwise 50 5 0.2 –biallelic-only –allow-extra-chr –allow-no-sex –noweb). Bi-allelic SNPs were quality checked using SNPrelate (v1.22.0) (Zheng et al., 2012).

### Analysis of structural variants

Multi-sample structural variant analysis and annotation were performed using smoove (v 0.2.6).

### Functional annotation of bamboo gene models

Protein sequences predicted for the bamboo HiC genome assembly were downloaded from gigadb^6^ (Zhao et al., 2018). They were compared to the InterPro database using interproscan (v5.32-71.0). Matched InterPro entries, Gene Ontology (GO), and pathways were used to functionally annotate predicted proteins.

## Results and discussion

### Whole genome resequencing

Whole genome re-sequencing analysis of the 25 bamboo genotypes, including 8 species of the *Phyllostachys* genus and 18 ecotypes of *Ph. edulis*, generated a total of 1,497.8 Gb of clean sequencing data, with an average of 59.9 Gb for each genotype after filtering out the low-quality reads. The mean sequencing depth for each accession was 28.52 (Supplementary Table 1). The mapping rate to the reference Moso bamboo genome ranged from 70.1 to 86.5%, with an average of 84.11 and 76.38% for *Ph. edulis* and other *Phyllostachys* species, respectively, demonstrating that the resequencing covered most of the genome not only in *Ph. edulis* but also in the other species for *Phyllostachys* genus (Supplementary Table 1).

### Variant calling, characterization, and annotation

The high-quality reads were mapped to the reference genome and multi-sample joint variant calling identified an average of 15,131,592 SNPs, 647,155 Insertions, and 1,015,313 Deletions in *Ph. edulis* genotypes (Supplementary Table 2). Whereas, the mean number of variants identified in the other *Phyllostachys* species is significantly higher due to the genetic distance between those species and the reference genome (*Ph. edulis*), highlighting the higher number of genomic and structural variations among the species (Supplementary Table 2). The SnpEff program was elaborated to annotate and evaluate the potential effects of the identified variants could have on the genes (Cingolani et al., 2012). According to their impact the SNPs and InDels were classified into four classes (High, Moderate, Low, and Modifier) (Tables 1, 2). As expected, most SNPs and InDels are modifiers, 98.6 and 99.2%, respectively. Interestingly, we identified 98,700 (0.44%) InDels and 63,341 SNPs (0.036%) with disruptive impact on the protein (Tables 1, 2 and Supplementary Table 3). These findings are in accordance with previous similar studies on whole genome resequencing of *Prunus* species, *Coffea arabica*, and Moso bamboo (Shirasawa et al., 2017; Xanthopoulou et al., 2020; Mekbib et al., 2022). Usually, the gene function is not affected by the synonymous SNPs; however, the moderate (possible non-synonymous substitution) and the high impact list of variants within *Ph. edulis* species but also within *Phyllostachys* genus consists a valuable source of potential candidate genes related to the phenotypic variation (Supplementary Dataset Files 1, 2).^7^ Similarly, although the distribution of both SNPs and InDels is more abundant in the non-coding regions (intergenic, downstream, and upstream of genes), there is a limited but a highly significant number of both variants detected in 5’UTR, 3’UTR, introns, and exons (Supplementary Tables 4, 5). Most of the single nucleotide changes can be classified as transitions in both *Ph. edulis* (11,097,172 SNPs) and the other *Phyllostachys* species (50,381,775 SNPs), with a transition/transversion ratio (Ts/Tv ratio) of 3.8851 across all the sequenced genotypes (Supplementary Table 6). The insertion/deletion ratio is 1.03 among the *Phyllostachys* species and 0.63 within the *Ph. edulis* strains (Supplementary Table 2).

**TABLE 2:** Effects of the identified INDELs on genes.

Impact class	Count	Percent (%)
High	98,700	0.438
Moderate	22,943	0.102
Low	65,743	0.291
Modifier	22,368,190	99.169

### Genetic differentiation and phylogenetic analysis

**FIGURE 3:**
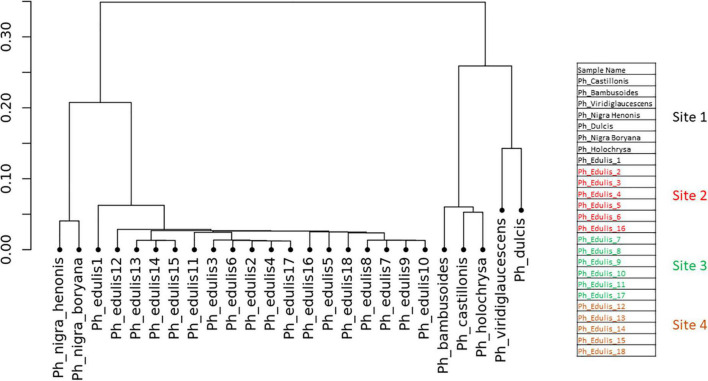
Hierarchical cluster dendrogram based on pairwise identity-by-state (IBS) values from SNP data.

**FIGURE 4:**
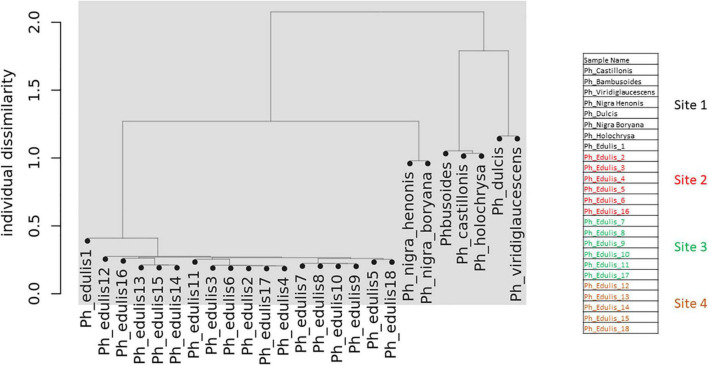
Dendrogram based on dissimilarity matrix of Euclidean distances from SNP data.

To explore the relationships among the *Phyllostachys s*pecies and *Ph. edulis* genotypes, a phylogenetic analysis was performed. Two hierarchical cluster dendrograms were generated based on pairwise identity-by-state (IBS) values and based on dissimilarity matrix of Euclidean distances from SNP data for all samples (Figures 3, 4). Both dendrograms clustered all the samples in two main groups. The first group with a sub-cluster including all the *Ph. edulis* genotypes and the other subcluster that consisted of the two strains of *Phyllostachys nigra*. The second group, also, is subdivided into two clusters that separate *Ph. bambusoides* strains from the *Ph. viridiglaucescens* and *Phyllostachys dulcis* species.

Despite the grouping of *Ph. edulis* plants, both dendrograms prove that these plants were not clones of the same rhizome and indeed they are progenies of seedlings derived from various collections of *Ph. edulis* in the four sites at Anduze in France (Figures 3, 4).

### Phenotypic variation of splitting strength trait

Bamboo can be considered a fiber-reinforced material with parenchyma cells as matrix and fibers as reinforcement. The fiber/matrix ratio of a culm cross-section and the distribution through the culm wall greatly affects the mechanical properties of bamboo (Akinbade et al., 2019). It has been shown that the fiber distribution across the culm section is not uniform; in fact, there is a higher fiber/matrix ratio in the outer part of the culm wall. Moreover, the fiber ratio varies along the culm height as shown by Anokye et al. (2014). This variation in fiber ratio and distribution along the height means that the mechanical properties need to be investigated at multiple cross-sections. Therefore, three samples are required by the ISO 22157:2019, 2019 standard to fully characterize the mechanical properties of a single bamboo culm. According to the same standard, the three samples must be taken from specific locations from the bottom (B), middle (M), and top (T) of each culm. In the culms that were collected, the B, M, and T specimens were taken from the same position in the culm with an accuracy on the order of ±10 cm to avoid the nodes.

The mechanical properties of bamboo have been extensively studied at different size scales (Jain et al., 1992; Chung and Yu, 2002; Yu et al., 2011; Wang et al., 2012; Chen et al., 2015). However, it is splitting (i.e., tension normal to the fibers) that dominates failure in structural applications (Janssen, 1981; Arce-Villalobos, 1993; Sharma et al., 2008). Splitting strength also influences the behavior of bamboo connections (Paraskeva et al., 2019 and references therein). Mitch et al. (2010) proposed a test to characterize the splitting strength of bamboo culms. The proposed splitting test uses a full culm section, thereby eliminating some of the complexities of partial culm tests, and it is adopted by ISO (ISO 22157:2019, 2019) (Supplementary Figure 1). Sharma et al. (2013) suggested a simplified and easy-to-perform in the field alternative to the splitting test proposed by Mitch et al. (2010). In this study, the splitting strength of bamboo was determined following the testing protocol of ISO 22157:2019.

**FIGURE 5:**
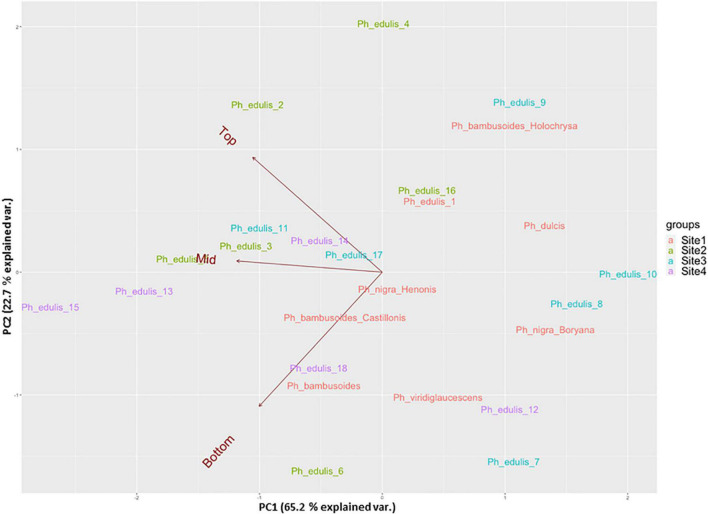
Principal component analysis (PCA) in a biplot of splitting strength trait of the three parts of bamboo culm (variables: dark red) of 25 bamboo genotypes collected from 4 sites.

**FIGURE 6:**
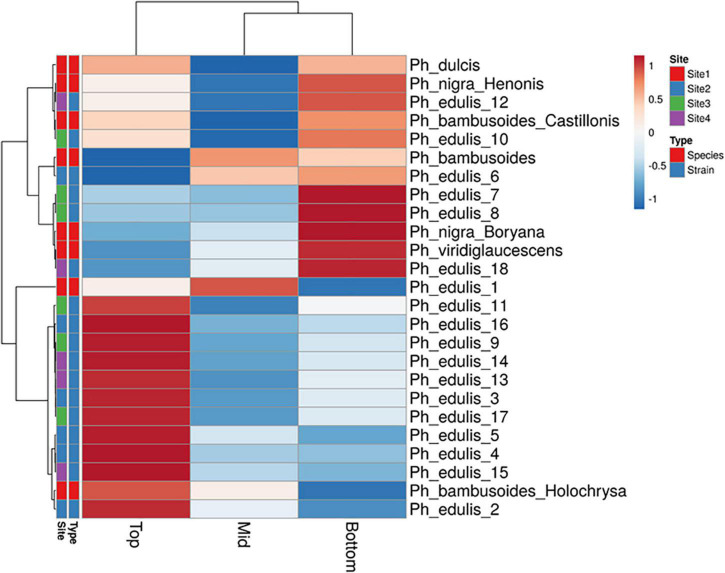
Hierarchical cluster analysis (HCA) based on Pearson’s correlation and average linkage with heat map representation of splitting strength trait. Rows are centered; unit variance scaling is applied to rows.

**FIGURE 7:**
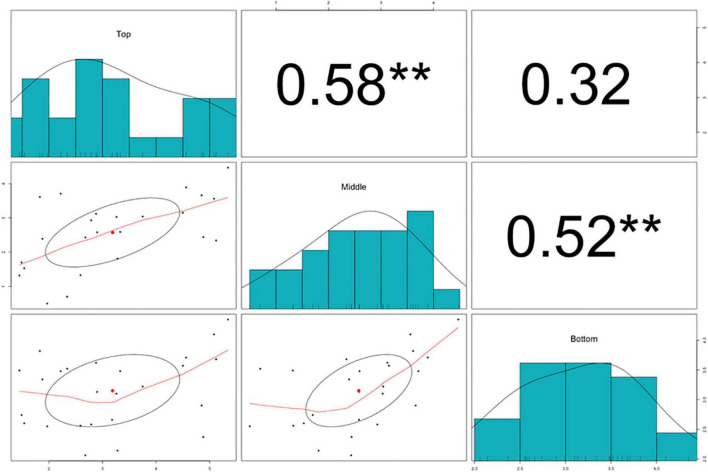
Pearson’s correlation analysis, scatter plots, and density histograms on the splitting strength of the bottom, middle, and top part of the bamboo culm. Significant correlations are denoted with asterisks at significance level **p < 0.01.

The splitting strength from the bottom, middle, and top of each culm was phenotyped for a total of 25 genotypes that include 8 *Phyllostachys* species and 18 accessions of *Ph. edulis* (Table 3). The phenotypic variation among the accessions was further analyzed by performing a Principal Component Analysis (PCA) and a Hierarchical Cluster Analysis (HCA) based on Pearson’s correlation (Figures 5, 6). The PCA was performed using all phenotyped traits (bottom, middle, and top of each culm). The first principal component explained a major part of the total variance (65.2%) that effectively differentiates most of the samples. The second principal component seems to differentiate the samples according to the splitting strength of the three parts of the bamboo culm, based on the projected variables of the PCA biplot, explaining 22.7% of the total variance (Figure 5). In addition to PCA and in order to explore the similarities as well as the differences of each sample between the splitting strength of the different parts of bamboo culm, a clustered heatmap analysis was performed (Figure 6). The clustering analysis grouped most of the *Ph. edulis* genotypes (12/18) and *Ph. bambusoides* “Holochrysa” together due to the similar splitting strength of the upper part of the bamboo culm. The similar phenotypic profiles of the bottom part of the bamboo culm clustered together the six *Ph. edulis* genotypes and the other *Phyllostachys* species (Figure 6). The distinct phenotypic profiles and the differential splitting strength between the top and the bottom part of the bamboo culm that is observed in the clustering analysis confirm the PCA result. Pearson’s correlation analysis showed that although there is a positive correlation between the top or the bottom with the middle part of the bamboo culm, there is a smaller correlation of the splitting strength between the top and the bottom part (Figure 7).

**TABLE 3:** Splitting strength (f_*t*,90_) of bamboo culms according to ISO 221757:2019 (MPa).

Sample	Site	Top	Mid	Bottom
Ph_Castillonis	Site1	3.33	2.59	3.48
Ph_Bambusoides	Site1	2.22	3.71	3.48
Ph_Viridiglaucescens	Site1	1.88	2.39	3.34
Ph_Nigra_Henonis	Site1	2.9	2.58	3.13
Ph_Dulcis	Site1	2.59	1.33	2.56
Ph_Nigra_Boryana	Site1	1.49	1.7	2.74
Ph_Holochrysa	Site1	2.68	2.43	2.06
Ph_Edulis_1	Site1	2.79	2.93	2.58
Ph_Edulis_2	Site2a	4.85	3.66	2.9
Ph_Edulis_3	Site2a	4.5	3.15	3.57
Ph_Edulis_4	Site2a	4.88	2.44	2.37
Ph_Edulis_5	Site2a	4.56	3.89	3.71
Ph_Edulis_6	Site2a	1.83	3.61	3.82
Ph_Edulis_7	Site2b	1.45	1.32	3.49
Ph_Edulis_8	Site2b	1.54	1.53	2.6
Ph_Edulis_9	Site2b	3.28	1.81	2.14
Ph_Edulis_10	Site2b	1.97	0.5	2.55
Ph_Edulis_11	Site2b	5.12	2.34	3.68
Ph_Edulis_12	Site2c	2.34	0.7	3.52
Ph_Edulis_13	Site2c	5.08	3.56	4.1
Ph_Edulis_14	Site2c	3.75	3.04	3.22
Ph_Edulis_15	Site2c	5.34	4.47	4.35
Ph_Edulis_16	Site2a	3.18	2.58	2.66
Ph_Edulis_17	Site2b	3.26	3.03	3.1
Ph_Edulis_18	Site2c	2.88	3.12	3.62

### Genome wide association study of splitting strength trait

A Genome Wide Association Study (GWAS) was performed aiming at identifying associations between genetic variations and the structural property trait of splitting strength in bamboo plants of *Phyllostachys* genus. The association panel comprised 8 *Phyllostachys* species and 18 natural accessions of *Ph. edulis*. The reasons that we had included in our association analyses not only accessions from the same species but also other species was to increase the genetic variation of our panel but also to find common genomic regions across the genus of *Phyllostachys* that are associated with such an important structural property. The availability of the Moso bamboo reference genome that belongs to the genus *Phyllostachys* and the generation of population-based high-coverage sequence data can significantly assist the mapping of genomic variants that are associated with the structural property traits. Before the analysis, SNPs and InDels with MAF <0.05 and more than 95% missingness were included in the association analyses, leaving a total number of 3,399,697 SNPs and 462,821 small InDels.

**FIGURE 8:**
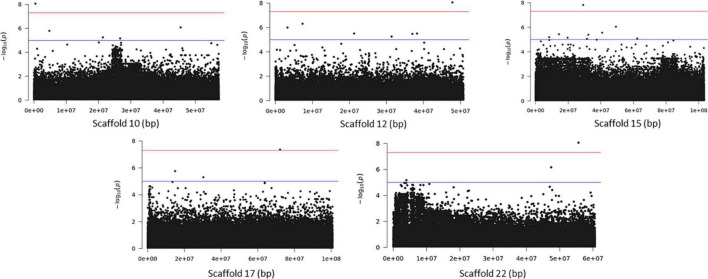
Genome Wide Association Analysis on the top part of bamboo. Manhattan plots of scaffolds harboring SNPs with *p* ≤ 10^–7^.

**FIGURE 9:**
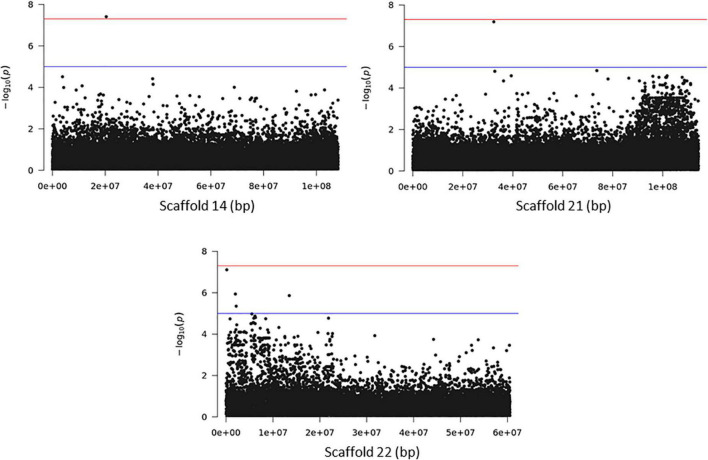
Genome Wide Association Analysis on the top part of bamboo. Manhattan plots of scaffolds harboring INDELs with *p* ≤ 10^–7^.

Association analysis with the SNP dataset identified 5 variants on 5 scaffolds, significantly associated (corrected *p* < 10^–7^) with splitting strength in the top part of bamboo culm (Figure 8 and Supplementary Table 7). We identified 9 candidate genes within an interval of 50 kb up- and downstream of the SNP variant. Among those genes, it is noteworthy to mention the *PH02Gene21516.t2* (Ma et al., 2021) that encodes a plant-specific YABBY transcription factor (TF) (Supplementary Table 7). These small zinc finger TFs are associated with plant morphogenesis, the development and polarity of lateral plant organs (Bowman, 2000; Kumaran et al., 2002), and biotic/abiotic stress (Yamada et al., 2011). Another interesting gene in the list is the *PH02Gene36721.t1*, which encodes a sugar transporter protein (Supplementary Table 7). The role of sugar in every aspect of plant development and growth is central. The mature basal elongated nodes and stems accumulate the highest amount of sucrose (Rae et al., 2005; Bihmidine et al., 2015), although it is believed that this is primarily driven by the termination of internode elongation (Hoffmann-Thoma et al., 1996; Julius et al., 2017). Recently Zhang et al. (2021) studied the association of starch storage and metabolism with the growth of bamboo culms, highlighting the important role of the bamboo shoot as the main organ for the storage of starch. The content of soluble sugars is higher in the upper part compared to the basal part but this is gradually decreased during development (Zhang et al., 2021). Therefore, the gene *PH02Gene36721.t1* shall be a potential candidate gene that may play a role in the tensile strength of the top part of the bamboo culm. In addition to the SNPs, we employed the small InDels for separate association analysis. Interestingly, we identified 1 significant locus (corrected *p* < 10^–7^) at the bottom of scaffold 14 and 2 more loci at scaffolds 21 and 22 that are very close to the threshold, and found that they are also associated with the tensile strength of the top part of the bamboo culm (Figure 9). At the locus of scaffold 21, we identified the gene *PH02Gene31570.t1* that encodes the transposase-derived transcription factor FAR-RED IMPAIRED RESPONSE1 (FAR1) (Figure 9 and Supplementary Table 8). This TF has been extensively studied for its crucial functions in plant development and growth (Ma and Li, 2018) and might also play a role in the studied structural property of bamboo.

**FIGURE 10:**
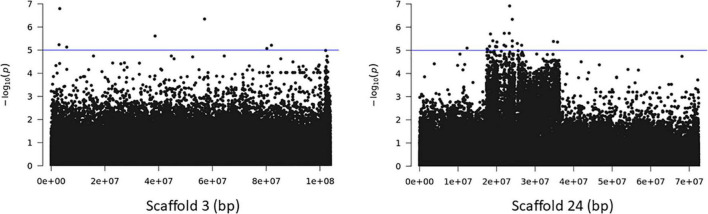
Genome Wide Association analysis of the trait middle part of bamboo. Manhattan plots of scaffolds harboring SNPs with *p* ≤ 10^–5^.

**FIGURE 11:**
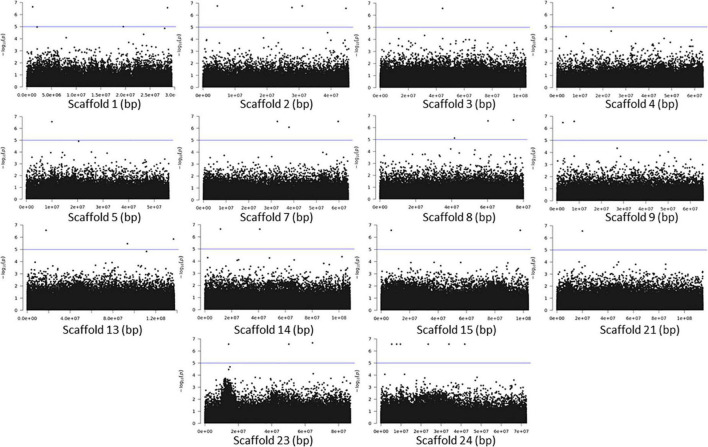
Genome Wide Association Analysis on the bottom part of bamboo. Manhattan plots of scaffolds harboring SNPs with *p* ≤ 10^–5^.

**FIGURE 12:**
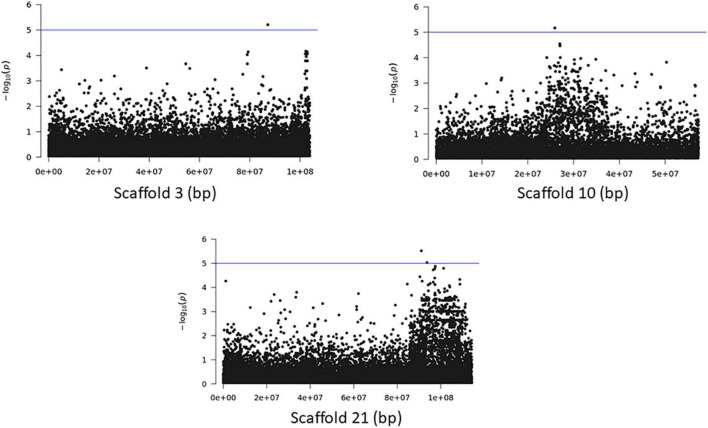
Genome Wide Association analysis of the trait middle part of bamboo. Manhattan plots of scaffolds harboring INDELs with *p* ≤ 10^–5^.

The association analyses using either SNPs or small InDels for the middle and bottom part did not result in any variant over the threshold, however, we applied the same process as before for the identification of candidate genes by studying only the loci with *p* < 10^–5^ (Figures 10–12, Supplementary Figure 1, and Supplementary Tables 7, 8). The SNP association analyses of the splitting strength of the middle and bottom part of bamboo culm identified 36 variants on 16 scaffolds with 61 potential candidate genes (Figures 10, 11 and Supplementary Table 7). A plethora of bamboo genes that encode transcription factors were identified within those 36 loci. Interestingly, we have identified genes that are related to ethylene and plant tissue senescence or aging such as *PH02Gene27779.t1*, *PH02Gene02347.t1*, and *PH02Gene36292.t1* (Supplementary Table 7). Genes that play an important role in plant aging might be possibly related to the structural properties of such woody tissue as bamboo culm. The small InDel association analyses for splitting strength of the lower parts of bamboo culm identified 6 variants on 5 scaffolds with 14 potential candidate genes, including genes with unknown functions and genes that encode proteins with kinase, FAD/NAD(P)-binding, and SAWADEE domains (Figure 12, Supplementary Figure 1, and Supplementary Table 8). The presence of significant single SNPs or InDels in the association analyses, without other neighboring significant SNPs/InDels has been identified in previous studies as well (Leforestier et al., 2015; Song et al., 2018). The most common reason can be that the significant SNP is in low LD (r^2^) with around variants or the minor allele frequency of that SNP is small. Nonetheless, these loci and candidate genes need to be further investigated. This step is crucial to confirm an association and rule out false positives. Thus, future work, examining the function of candidate genes through molecular functional studies, will be needed to understand the genetic architecture of these complex structural property traits and the causal relationships between the phenotype and the responsible genotype.

## Conclusion

This is the first report of whole genome genetic variation characterization between *Ph. edulis* strains but also among other important *Phyllostachys* species. Additionally, an important trait, the splitting strength that is strongly related to the structural performance of bamboo was phenotyped and it was associated with genomic regions that include several important candidate genes that can be used for population genetic studies and fine genetic association analyses. Also, we identified a large set of molecular markers providing key genomic resource information for several *Phyllostachys* species that will assist and improve further the efficiency of future breeding efforts. Therefore, this study provides the first important step in understanding the genetic architecture of a complex structural property trait, such as the splitting strength, in bamboo.

## Data availability statement

The datasets presented in this study can be found in online repositories. The names of the repository/repositories and accession number(s) can be found in the article/Supplementary material.

## Author contributions

MV and CB conceived and designed the study and obtained the funding. LD, MV, and CB obtained the specimens from the field. LD performed the structural testing and drafted the part related to structural engineering. CB extracted the DNA and drafted the part related to biology. MV edited the manuscript. All authors contributed to the article and approved the submitted version.

## Conflict of interest

The authors declare that the research was conducted in the absence of any commercial or financial relationships that could be construed as a potential conflict of interest.

## Publisher’s note

All claims expressed in this article are solely those of the authors and do not necessarily represent those of their affiliated organizations, or those of the publisher, the editors and the reviewers. Any product that may be evaluated in this article, or claim that may be made by its manufacturer, is not guaranteed or endorsed by the publisher.

## Footnotes

1
https://www.arb-silva.de/

2
https://www.ncbi.nlm.nih.gov/tools/vecscreen/univec/

3
https://www.ncbi.nlm.nih.gov/refseq/

4
http://gigadb.org/dataset/view/id/100498/File_page/2

5
http://vcftools.sourceforge.net

6
https://ftp.cngb.org/pub/gigadb/pub/10.5524/100001_101000/100498/Gene_PEP/Bamboo.Hic.pep.gz

7
https://www.research-collection.ethz.ch/handle/20.500.11850/543942

## Supplementary material

Supplementary Figure 1 Genome Wide Association Analysis on the bottom part of bamboo. Manhattan plots of scaffolds harboring INDELs with *p* ≤ 10^–5^.

## Appendix A

**APPENDIX TABLE A1:** Dimensions of all specimens.

			Bottom					Middle					Top		
			
Specimen	D	d	δ	L	*f*_*t*,90_	D	d	δ	L	*f*_*t*,90_	D	d	δ	L	*f*_*t*,90_
*Ph. Bambusoides _Castillonis*	61.15	22.00	7.70	83.15	3.48	59.55	22.00	5.00	81.55	2.59	36.75	12.00	3.60	48.75	3.33
*Ph. Bambusoides*	67.60	22.00	8.20	89.60	3.48	66.10	22.00	3.20	88.10	3.71	31.80	12.00	2.80	43.80	2.22
*Ph. Viridiglaucescens*	57.40	22.00	9.00	79.40	3.34	56.80	22.00	5.10	78.80	2.39	37.95	12.00	4.10	49.95	1.88
*Ph. Nigra Henonis*	65.25	22.00	7.10	87.25	3.13	61.40	22.00	5.30	83.40	2.58	39.30	12.00	3.90	51.30	2.90
*Ph. Dulcis*	68.80	22.00	6.65	90.80	2.56	62.30	22.00	4.15	84.30	1.33	42.00	12.00	3.00	54.00	2.59
*Ph. Nigra Boryana*	68.25	22.00	7.50	90.25	2.74	61.05	22.00	6.80	83.05	1.70	32.05	12.00	4.10	44.05	1.49
*Ph. Bambusoides _Holochrysa*	85.75	22.00	9.30	107.75	2.06	81.15	22.00	5.35	103.15	2.43	47.80	22.00	3.85	69.80	2.68
*Ph. Edulis1*	79.95	22.00	9.65	101.95	2.58	69.30	22.00	6.75	91.30	2.93	45.40	12.00	4.60	58.15	2.79
*Ph. Edulis2*	74.05	22.00	8.60	96.05	2.90	49.50	22.00	4.10	71.50	3.66	23.20	12.00	2.10	35.20	4.85
*Ph. Edulis3*	76.10	22.00	9.00	98.10	3.57	55.80	22.00	4.50	77.80	3.15	29.40	12.00	3.00	41.40	4.50
*Ph. Edulis4*	80.05	22.00	9.10	102.05	2.37	64.85	22.00	5.10	86.85	2.44	35.65	12.00	3.20	47.65	4.88
*Ph. Edulis5*	68.25	22.00	8.50	90.25	3.71	48.60	22.00	3.90	70.60	3.89	36.60	12.00	3.10	48.60	4.56
*Ph. Edulis6*	66.75	22.00	8.90	88.75	3.82	51.70	22.00	4.20	73.70	3.61	29.00	12.00	2.30	41.00	1.83
*Ph. Edulis7*	63.65	22.00	8.60	85.65	3.49	56.50	22.00	5.40	78.50	1.32	31.35	12.00	3.10	43.35	1.45
*Ph. Edulis8*	64.75	22.00	8.90	86.75	2.60	56.75	22.00	5.20	78.75	1.53	36.10	12.00	3.20	48.10	1.54
*Ph. Edulis9*	61.40	22.00	8.90	83.40	2.14	50.25	22.00	4.40	72.25	1.81	33.05	12.00	3.00	45.05	3.28
*Ph. Edulis10*	63.40	22.00	7.30	85.40	2.55	58.25	22.00	5.20	80.25	0.50	33.70	12.00	2.20	45.70	1.97
*Ph. Edulis11*	65.80	22.00	9.10	87.80	3.68	46.85	22.00	4.20	68.85	2.34	27.90	12.00	3.00	39.90	5.12
*Ph. Edulis12*	72.95	22.00	9.90	94.95	3.52	58.25	22.00	5.30	80.25	0.70	32.15	12.00	2.10	44.15	2.34
*Ph. Edulis13*	64.70	22.00	7.40	86.70	4.10	52.05	22.00	4.00	74.05	3.56	33.45	12.00	2.50	45.45	5.08
*Ph. Edulis14*	68.80	22.00	9.20	90.80	3.22	55.85	22.00	4.30	77.85	3.04	33.95	12.00	2.90	45.95	3.75
*Ph. Edulis15*	66.00	22.00	7.90	88.00	4.35	50.45	22.00	4.20	72.45	4.47	30.25	12.00	2.80	42.25	5.34
*Ph. Edulis16*	102.30	22.00	10.25	124.30	2.66	83.20	22.00	6.85	105.20	2.58	64.30	22.00	5.28	85.05	3.18
*Ph. Edulis17*	87.85	22.00	9.50	109.85	3.10	71.75	22.00	6.35	93.75	3.03	51.80	22.00	4.30	71.35	3.26
*Ph. Edulis18*	92.90	22.00	10.55	114.90	3.62	71.55	22.00	6.00	93.55	3.12	43.80	12.00	3.55	54.05	2.88
