# Supplementary figures and images for: Study of genetic variation and its association with tensile strength among bamboo species through whole genome resequencing
Source: Front Plant Sci. 2022 Jul 27;13:935751. doi: 10.3389/fpls.2022.935751 (PMC9365670; doi:10.3389/fpls.2022.935751)

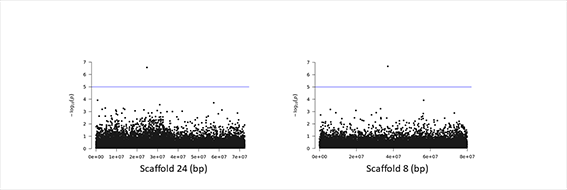

Supplement: Supplementary Figure 1 — Genome Wide Association Analysis on the bottom part of bamboo. Manhattan plots of scaffolds harboring INDELs with p ≤ 10–5. [file Image_1.TIF]
